# Complete response to neoadjuvant chemoradiotherapy in rectal cancer is associated with RAS/AKT mutations and high tumour mutational burden

**DOI:** 10.1186/s13014-021-01853-y

**Published:** 2021-07-13

**Authors:** Joanne D. Stockton, Louise Tee, Celina Whalley, Jonathan James, Mark Dilworth, Rachel Wheat, Thomas Nieto, S-CORT Consortium, Ian Geh, João D. Barros-Silva, Andrew D. Beggs

**Affiliations:** 1 Surgical Research Laboratory, Institute of Cancer and Genomic Science, University of Birmingham, Vincent Drive, Birmingham, B15 2TT UK; 2 University Hospitals Birmingham NHS Foundation Trust, Birmingham, UK

## Abstract

**Background:**

Pathological complete response (pathCR) in rectal cancer is beneficial, as up to 75% of patients do not experience regrowth of the primary tumour, but it is poorly understood. We hypothesised that the changes seen in the pre-treatment biopsies of pathCR but not seen in residual tumour after chemoradiotherapy were the determinants of responsiveness.

**Methods:**

Two groups of patients with either complete response (pathCR group, N = 24) or no response (poor response group, N = 24) were retrieved. Pre-treatment biopsies of cancers from these patients underwent high read depth amplicon sequencing for a targeted panel, exome sequencing, methylation profiling and immunohistochemistry for DNA repair pathway proteins.

**Results:**

Twenty four patients who underwent pathCR and twenty-four who underwent poor response underwent molecular characterisation. Patients in the pathCR group had significantly higher tumour mutational burden and neoantigen load, frequent copy number alterations but fewer structural variants and enrichment for driver mutations in the PI3K/AKT/mTOR signalling pathway. There were no significant differences in tumour heterogeneity as measured by MATH score. Methylation analysis demonstrated enrichment for hypomethyation in the PI3K/AKT/mTOR signalling pathway.

**Discussion:**

The phenomenon of pathCR in rectal cancer may be related to immunovisibility caused by a high tumour mutational burden phenotype. Potential therapy resistance mechanisms involve the PI3K/AKT/mTOR signalling pathway, but tumour heterogeneity does not seem to play a role in resistance.

**Supplementary Information:**

The online version contains supplementary material available at 10.1186/s13014-021-01853-y.

## Introduction

Rectal cancer is a common malignancy [1], with approximately 11,000 cases per year in the UK [2]. Treatment typically consists of excisional surgery [3] with neoadjuvant therapy if the cancer is locally advanced, consisting of either short course radiotherapy [4] (25 Gy in 5 fractions over 1 week) with surgery the following week, or long course neo-adjuvant chemoradiotherapy (nCRT) [5] (45–50 Gy in 25 fractions over 5 weeks, with synchronous iv 5-FU or oral capecitabine) with surgery 6–10 weeks later. The former regimen usually demonstrates little, if any tumour regression, but if it does occur, is associated with more favourable prognosis, and the latter regimen can lead to significant tumour shrinkage and downstaging, with pathological complete response (pathCR, defined as complete regression of tumour in the resection specimen) observed in approximately 10–15% of patients [6]. Multiple investigators have shown higher rates of response [7, 8] with higher radiotherapy doses, with maximum pCR rates of 25–30%. The current standard of care within the United Kingdom for locally advanced rectal cancer is long course chemoradiotherapy, however the recent development of the concept of “total” neo-adjuvant therapy (TNT), whereby consolidation chemotherapy is given after chemoradiotherapy, increased pCR rates from 15 to 25% in the consolidation group, acting as an additional therapeutic option in these patients which may lead to its introduction as standard of care [9]. Short course radiotherapy with a delay to surgery [10], or with neoadjuvant chemotherapy in the RAPIDO trial [11], has also been shown to be of benefit and demonstrates the critical nature of the sequencing and timing of treatment in order to maximise response.

Clinical complete response (CCR) is defined as the absence of tumour on imaging and/or clinical examination, but does not definitively exclude residual tumour, which requires a resection specimen to confirm. CCR is well correlated with pathCR and, this could allow routine use of a watch and wait strategy. In addition to its role as an indicator of potential cure [12] (defined as > 5 years recurrence free), path CR could be used to delay excisional surgery [13] or allowing organ preservation surgery [14], such as TEMS or TAMIS [15].

The molecular drivers of pathCR are unclear [16, 17], but they are thought to relate to factors that promote radiotherapy- and chemotherapy-related tumour cell killing. For example, rectal cancers exist within a low oxygen tension environment [18] leading to intrinsic resistance. As radiotherapy induces the formation of oxygen derived free radicals, which cause tumour cell death by direct DNA damage, a low oxygen environment leads to fewer available oxygen free radicals, leading to lower cell death. Another hypothesised mechanism underlying differences pathCR is variation in DNA repair. Ionising radiation [19] induces a DNA double strand break (DSB), which is then repaired either by homologous recombination (HR), where a sister chromatid is used to repair the defect, or non-homologous end joining (NHEJ), where a complex repair mechanism including *BRCA*, *ATM*, *ERCC5* and others contribute to a DNA repair complex that re-joins the damaged segments of DNA. There is evidence from previous studies that aberrant functioning of the NHEJ pathway is associated with a longer survival, presumably as a consequence of better response in radiotherapy [20], however the data concerning the role of NHEJ and the response to radiotherapy is unclear, with studies giving conflicting reports of its relationship with radiation response [21]. Lal et al. [22] also showed that the immune context of a tumour depended on genomic factors, with *KRAS* mutation, CMS2 or CMS3 classification all being independently associated with a reduced immune infiltration. This has implications for responsiveness to therapy, as a large proportion (40%) of colorectal cancer has *KRAS* mutation, and immunovisibility of the tumour is essential for a good response to neoadjuvant therapy.

Tumour heterogeneity undoubtedly also plays a role in determining pathCR [16, 23], as does immunogenicity caused by the formation and expression of clonal neoantigens. Rectal cancers have little hypermutation and therefore rates of immunovisibility are low. Whilst Akiyoshi et al. [1] have recently shown that levels of clonal neoantigens are higher in patients undergoing a good response to neoadjuvant chemoradiotherapy, suggesting that hypermutation is important, a previous integrated molecular analysis of rectal cancer [16] found no key genomic features that correlated with resistance. This may be because of the significant heterogeneity of the datasets used in the analysis.

Owing to the uncertainty surrounding the precise mechanisms of sensitivity of rectal cancer to chemoradiotherapy, we aimed to study the phenomenon of pathCR in rectal cancer. We hypothesised that the genomic changes responsible for the phenomenon of pathCR would be seen uniquely in the pre-treatment biopsies of those undergoing a complete response, and not seen at all in the residual post-treatment specimens of patients who had undergone neo-adjuvant chemoradiotherapy and therefore would represent two divergent opposites to identify the phenomenon.

## Methods

### Patients

A prospective database of all patients undergoing neoadjuvant chemoradiotherapy for rectal cancer was used to identify patients. Ethical approval was obtained from the NW Research Ethics committee (ref 15/NW/0079). Patients who underwent long course chemoradiotherapy and achieved pathCR were identified, as determined by complete regression of the tumour, with absolutely no tumour cells remaining (Mandard grade 1/ TRG 1), on examination of the specimen by a Consultant Histopathologist, as opposed to minimal residual disease where several cells were allowable. All patients underwent long course chemoradiotherapy with either oral capecitabine (825 mg/m^2^) or infusional 5-flourouracil during a radiotherapy course of 45 Gy in 25 fractions over 35 days. Pre-treatment stage and post-treatment response (at 6 weeks after finishing treatment) was assessed using magnetic resonance imaging. Resection of the primary tumour then occurred as soon as possible after the 6 week MRI scan. The histopathology archives were then searched to find the pre-treatment endoscopic biopsies of these patients for downstream analysis. This was defined as the “pathCR cohort”. A randomly selected second cohort of rectal cancer patients was identified, who had no response to treatment or progressed whilst on treatment, as defined both by post-treatment MRI and Mandard grade 4 or 5. This was defined as the “non-responder cohort”. For the validation cohort, patients from work stream 3 of the Stratification in Colorectal cancer (S-CORT) were utilised, which represent a cohort of patients undergoing long course chemoradiotherapy for whom pre-treatment biopsy material was available. For germline DNA, representative normal tissue from the proximal resection margin of the discovery cohort was obtained for genomic analysis.

### Samples

Formalin fixed blocks were retrieved for these patients and cut into 4uM sections on frosted glass slides for needle macrodissection and immunohistochemistry. Pre-treatment specimens were all endoscopically obtained biopsies, whereas post-treatment specimens consisted of tumour blocks selected by a histopathologist as representative of the tumour. This consisted of the block whereby the H&E section showed maximum tumour content. A representative H&E section was used to target tumour cells for macrodissection to maximise tumour content for DNA extraction using a modified protocol of the Qiagen DNEasy kit (Qiagen). Eluted DNA was quantified using Nanodrop spectrophotometry (for contaminants) and Qubit fluorimetry (for concentration).

### Immunohistochemistry (IHC)

This was performed on 4uM slides as previously described using a Leica BondMax autostainer. IHC was performed against yH2AX (Abcam ref ab26350), ATM (ab36810), Ku70/80 (ab53126), MLH1 (ab92312), MSH2 (ab52266) and MSH6 (ab14204) and slides were then scanned on a Leica slide imaging platform. IHC was scored by the QuPath system [24]. Briefly, a single section was zoomed to 10 × view of a representative area of epithelium/tumour, calibration was performed according to the QuPath manual, either nuclei or membranous counting was set depending on the antibody and auto counting of DAB stained positive/negative cells was carried out. Staining was reported as a percentages of positive/negative cells.

### Genomics

Extracted genomic DNA was used for targeted amplicon resequencing using the Fluidigm 48 × 48 Access array. PCR amplicons covering APC, KRAS, BRAF, NRAS, FBXW7 and SOX7 (primer sequences available on request) were designed using Primer3 [25]. Primer specificity was checked using PrimerBLAST and UCSC in-silico PCR. Briefly, 20 ng of FFPE DNA was injected into the Access Array system with PCR primers and thermal cycled according to manufacturer’s specifications. Amplicons were then ligated to Illumina sequencing indexes & adapters and pooled and sequenced on an Illumina MiSeq to an average read depth of > 1000 × using a 100 bp PE sequencing strategy. For FFPE exome sequencing, a custom modification of the Illumina TruSeq Exome hybridisation kit was used. At least 300 ng of FFPE-derived DNA was prepared with the following modifications: Firstly, no size selection was performed after end repair and DNA fragments were amplified with 12 cycles of PCR. Enrichment was performed using a bead ratio of 0.8, then samples were combined into pools of 3 plex for coding exome (TruSeq exome, 45mb in size) probe hybridisation and subsequent clean up. 10 cycles of amplification were performed to enrich the final libraries which were then pooled into a final 12 plex library. Sequencing was performed on an Illumina NextSeq. For the S-CORT WS3 samples, a custom panel consisting of 61 oncogenes was sequenced to 500 × using Agilent SureSelect bait capture at the Wellcome Trust Sanger Institute. Methylation was interrogated using the Illumina HumanMethylation 450 array system. Between 100 and 250 ng of FFPE DNA was processed using the Illumina FFPE restore kit, and then hybridised to the HumanMethylation 450 array following the manufacturer’s instructions. Slides were washed and scanned on an Illumina iScan scanner.

### Imaging mass cytometry

Slide staining and CyTOF were performed on FFPE sections using methods as previously described [26]. CyTOF IMC data were analysed using an image-processing pipeline as described (https://github.com/BodenmillerGroup/imctools). Ilastik generated Probability probability maps and raw multi-channel files from each region of interest were analysed using CellProfiler and the Cytomapper R package (v3.12) [27, 28].

### Bioinformatics

For the amplicon resequencing, FASTQ files were trimmed (Trimgalore) and aligned to the GRCh38 reference genome using a standard pipeline using the BWA (v0.7.17-r1188) aligner [29]. Mutation calling was performed using FreeBayes (v1.0.0) [30]. For the samples from the SCORT consortium, alignment using BWA was carried out to the GRCh38 reference genome and mutation calling performed with Caveman (v1.14.0) and Pindel (v1.0) [31, 32]. For the exome sequencing analysis, FASTQ files were trimmed and aligned to the GRCh38 reference genome using an exome sequencing pipeline Isaac v4 aligner [33], Manta SV caller (v1.6.0) [34] and Canvas CNV (Canvas 1.40.0.1613 + master) [35] caller. Enrichment was determined by comparison to the Illumina TruSeq exome v1.2 BED file. Mutation calling was performed using Strelka 2 [36] and MuTect2 in tumour-normal subtraction mode. For both amplicon sequencing and exome analysis, significantly mutated genes were identified using Intogen (v1.0) [37], MutSigCV (v2.1) [38] and dNdScv (v.0.1.0) [39]. Mutational signatures were estimated using the MutationalPatterns (v3.0.1) [40] R package. Tumour mutational burden was estimating by annotation of the combined, overlapping variant calls from Mutect/Strelka2 to filter to non-synonymous variants (nsV). NsV were then divided by the TruSeq Exome panel size (45.3 Mb) to give a figure in mutations/mb.

For structural variant (SV) calls, Manta was used in tumour/normal mode on exome sequencing data and lists of structural variants outputted to VCF file. For copy number calls (CNV), Canvas was used in tumour normal mode and calls outputted as VCF files.

Clonality was determined by running superFreq (v1.0) [41], a cancer specific tumour exome caller and river plots were producing from this package. For neoantigen calls, tumour-normal subtracted VCF files produced with Strelka, GT fields were added via conversion of the SGT field (using a custom script), annotated with the variant effect predictor (VEP), filtered for indels and then analysed using PVacTools v2.0.0 against MHC Class I binding predictions [42]. Neoantigens with a “best” median IC of < 50 nmol L^−1^ were counted as binding neoantigens. HLA typing for each patient was determined with HLA-LA* (v1.0.1) on germline exome sequencing data [43].

For methylation, IDAT files were imported into R/Bioconductor and analysed on the ChAMP (v2.20.1) pipeline [44] using standard settings. iDAT files were imported, standardised to beta methylation values, then underwent SWAN normalisation. Normalised beta-values underwent differential methylation analysis using limma, DMR were called using dmrLasso. Pathway analyses were conducted in GProfiler2 [45].

For statistical analysis, Stata 15.1 was used, and all distributions plotted to check for a Gaussian distribution. Unpaired t-testing was set with a significance threshold of 0.05, and any missing values were imputed using the Stata impute commands.

## Results

### Samples

**Fig. 1:**
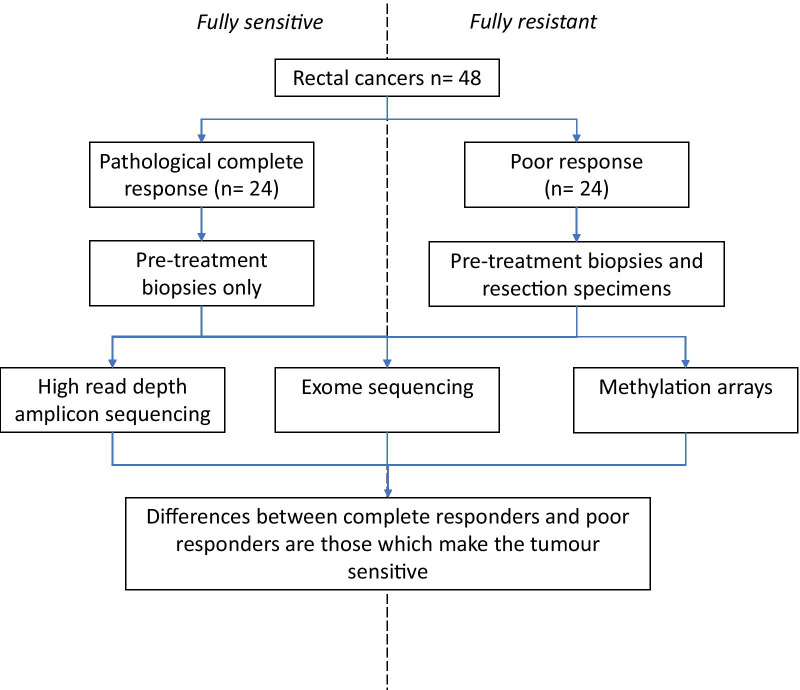
Flowchart of samples going through study

In total, there were 48 patients with samples available in the study (Fig. 1). In the pathCR group, 24 pre-treatment biopsies were available. In the post-treatment group there were 24 post-treatment samples that had a poor response to chemoradiotherapy as defined by histological tumour regression grade. The S-CORT WS3 cohort had 231 pre-treatment biopsies available for analysis with variable levels of response. Matched germline DNA was available for all samples apart from the S-CORT WS3 cohort.

### Sequencing metrics

All available samples successfully underwent amplicon sequencing and exome sequencing. For the amplicon sequencing average read depth was 1020 × (range 357–5210 ×). For the whole exome sequencing the average read depth was 99 × for tumour samples and 55 × for control normal. For methylation arrays all samples hybridised successfully.

### Mutational profiles

In the amplicon sequencing group, using treatment response as a classifier to select top significantly mutated genes correlated with response as defined by MutSigCV,were *PIK3CA* (p = 2.4 × 10^−9^), *FBXW7* (p = 8.68 × 10^−4^) and *PTEN* (p = 1.02 × 10^−3^). Neither dNdScV nor Intogen can classify mutations by co-variates therefore a Fisher exact test was used to compare the two groups (pathCR vs. non-responder) for the output of each caller. Using dNdScV, the top significantly mutated genes were *AKT1* (p_mis_ = 2.01 × 10^−3^), *FBXW7* (p_mis_ = 5.90 × 10^−3^), *FAM123B* (p_mis_ = 2.48 × 10^−2^) and *POLE* (p_mis_ = 1.32 × 10^−2^). Gene centric analysis with Intogen demonstrated recurrent mutations in *PIK3CA*, *PTEN*, *FBXW7* and *POLE*. Pathway analysis of the genes present in the pathCR group but not the non-responder group demonstrated 14 pathways (Table 1) being significantly over-represented in the dataset including hsa05210 (Colorectal Cancer, p = 1.88 × 10^−16^, q = 1.62 × 10^−14^), hsa05222 (Small cell lung cancer, p = 8.43 × 10^−12^, q = 3.63 × 10^−10^), and hsa04150 (mTOR pathway, p = 4.99 × 10^−10^, q = 1.43 × 10^−8^), all of which contain genes involved in the mTOR/AKT pathway.

**Table 1:** Intogen significantly enriched pathways

ID	Pathway	Z score	p value	Q value
hsa05210	Colorectal cancer	8.14	1.88E−16	1.62E−14
hsa05222	Small cell lung cancer	6.73	8.43E−12	3.63E−10
hsa04150	mTOR signalling pathway	6.10	4.99E−10	1.43E−08
hsa04310	Wnt signalling pathway	5.96	1.26E−09	2.71E−08
hsa05212	Pancreatic cancer	5.77	3.91E−09	6.60E−08
hsa00562	Inositol phosphate metabolism	5.71	5.37E−09	6.60E−08
hsa04070	Phosphatidylinositol signalling system	5.71	5.37E−09	6.60E−08
hsa04115	p53 signalling pathway	5.60	1.01E−08	1.09E−07
hsa05166	Human T-cell leukemia virus 1 infection	5.50	1.87E−08	1.79E−07
hsa05169	Epstein-Barr virus infection	4.99	2.96E−07	2.12E−06
hsa05162	Measles	4.99	2.96E−07	2.12E−06
hsa04210	Apoptosis	4.99	2.96E−07	2.12E−06
hsa05217	Basal cell carcinoma	4.66	1.56E−06	1.03E−05
hsa04120	Ubiquitin mediated proteolysis	4.59	2.16E−06	1.33E−05

For the exome sequencing data, analysis by MutSigCV using pathCR as a covariate revealed 1,412 genes that were significantly mutated (Additional file 1). The top genes were *HIVEP3*, *HS6ST3*, *KIAA1671*, *LRRC4C* and *ROBO2*. This was due to the preponderance of hypermutant samples within the pathway which lead to select of non-driver genes, and filtering would have removed samples. Pathway analysis by GProfiler demonstrated no enrichment of any KEGG pathways. Analysis by Intogen using revealed demonstrated 1620 genes significantly mutated (Additional file 1 as determined either by OncoDriveClust or OncoDriveFM. The top five genes were *CDC27*, *CTBP2*, *IGSF3*, *PABPC3* and *ZNF432*. Pathway analysis with Intogen demonstrated over-representation of focal adhesion (hsa04510, p = 2.25 × 10^−135^, q = 5.82 × 10^−133^), which contains within it the MAPK/PIK3K and Wnt signalling pathways; as the top rated pathway (Additional file 1). Analysis by dNdScV demonstrated 209 genes significantly mutated (p < 0.05), the top five of which were *ZNF717*, *MUC3A*, *APC*, *OR4C5* and *KRAS*.

Mutational signature analysis (version 3 single base substitution) was performed on the exome sequencing samples from the pathCR group. The top ranked signatures were signatures three, five and thirty. Signature three is postulated to be due to defective base repair due to faulty homologous recombination, signature five is due to the effects of transcription coupled nucleotide repair and signature 30 is due to a defect in base-excision repair due to inactivating mutations in *NTHL1* (Additional file 1).

**Fig. 2:**
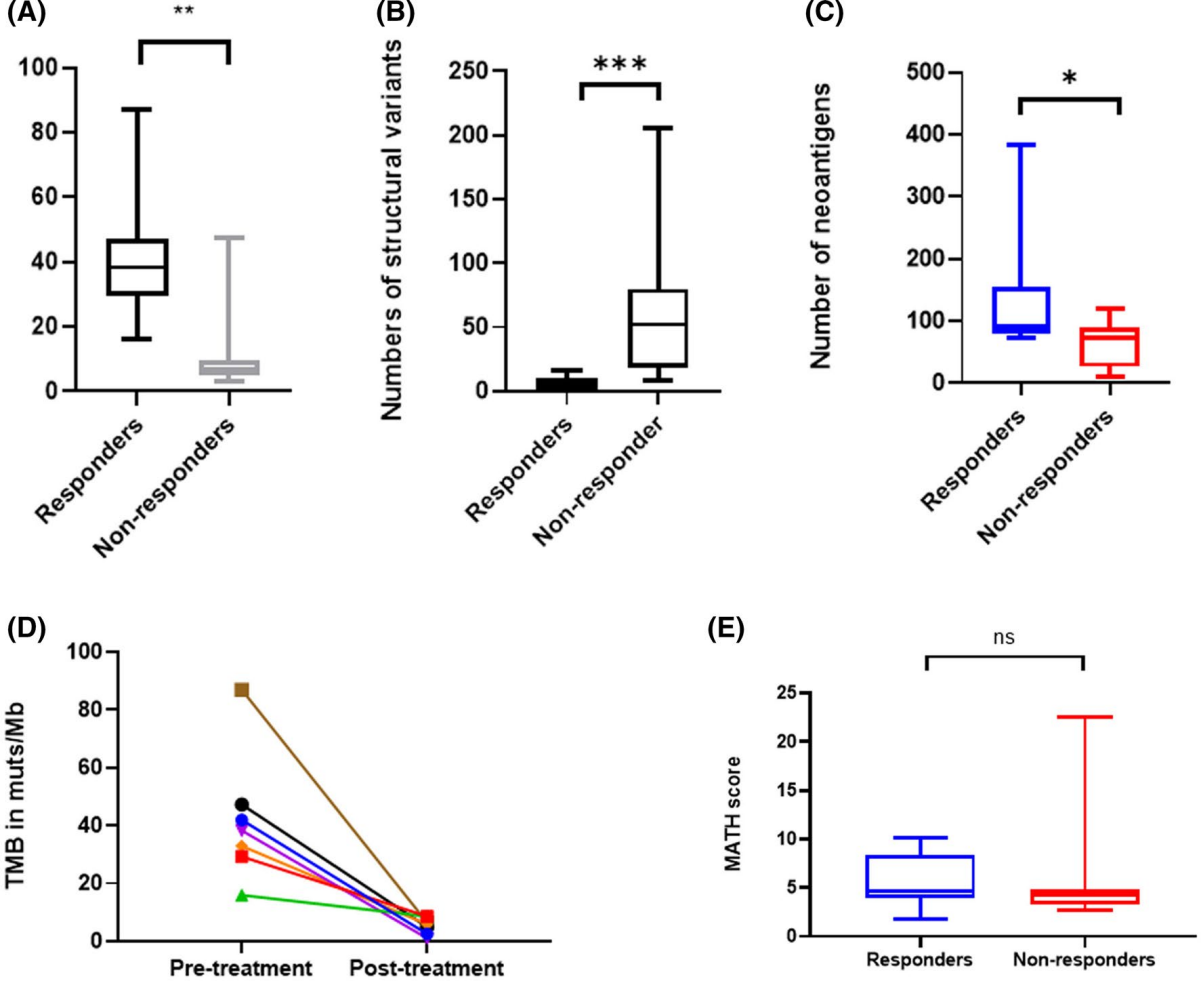
Differences in genomic characteristics between responders and non-responders. **A** Tumour mutational burden in mutations/megabase; **B** Structural variants per sample; **C** Numbers of neoantigens per sample; **D** Change in Tumour mutational burden in paired samples pre and post response; **E** MATH score per sample

Tumour mutation burden (TMB) was significantly higher in pre-treatment samples from pathCR patients than non-responders (exome data, median_pathCR_ = 38.28 muts/mb, range 15.93–86.95 *v* median_NR_ 7.27 muts/mb, range 3.08–47.3, p = 0.02 Wilcoxon, Fig. 2).

In order to validate the finding that tumour mutational burden seemed to correlate with response, we analysed data from the SCORT WS3 “Grampian” cohort consisting of pre-treatment biopsies (n = 231) from patients undergoing long course chemoradiotherapy. Total numbers of coding mutations were significantly (p = 0.036) greater in the “responder” (n = 35, mean 9.2 mutations) vs. “non-responder” (n = 196, mean 7.8 mutations) groups.

### Structural variants and copy number variation

**Fig. 3:**
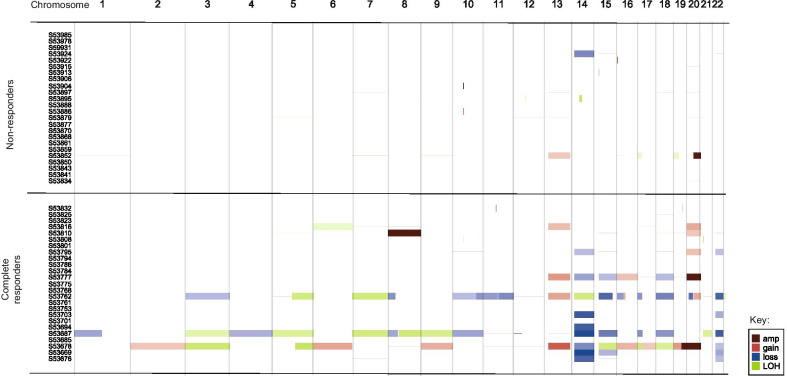
Copy number calls across cohort – ID on Y-axis

Copy number estimation was performed on all tumour: normal exome pairs successfully (Fig. 3). Three biopsy samples showed an extremely complex pattern of copy number gain and loss (with modal CN of 67, 52 and 66 respectively), which corresponded with complete pathological response to chemoradiotherapy. The median copy number of the pathCR group was 50 (IQR 45–66) and of the non-responder group was 46 (IQR 44–47, Wilcoxon Ranked sums p = 0.01). No recurrent copy number alterations were observed. In the poor response group, the copy number seen in the pre-treatment biopsy (median 45, IQR 44–49) did not significantly vary with the post treatment specimen (median 46, IQR 44–47, Wilcoxon Ranked sums p = 0.94).

Structural variant (SV) calling was performed on all tumour: normal exome pairs successfully. The median number of structural variants in the pre-treatment biopsies of the pathCR group was 4 (IQR 0–10) vs. 52 in non-responders group (IQR 18–80, p < 0.001, Fig. 2). There was no significant difference in the numbers of SV between the pre-treatment biopsies of the non-responders and the post-treatment specimens.

### Clonal evolution analysis

**Fig. 4:**
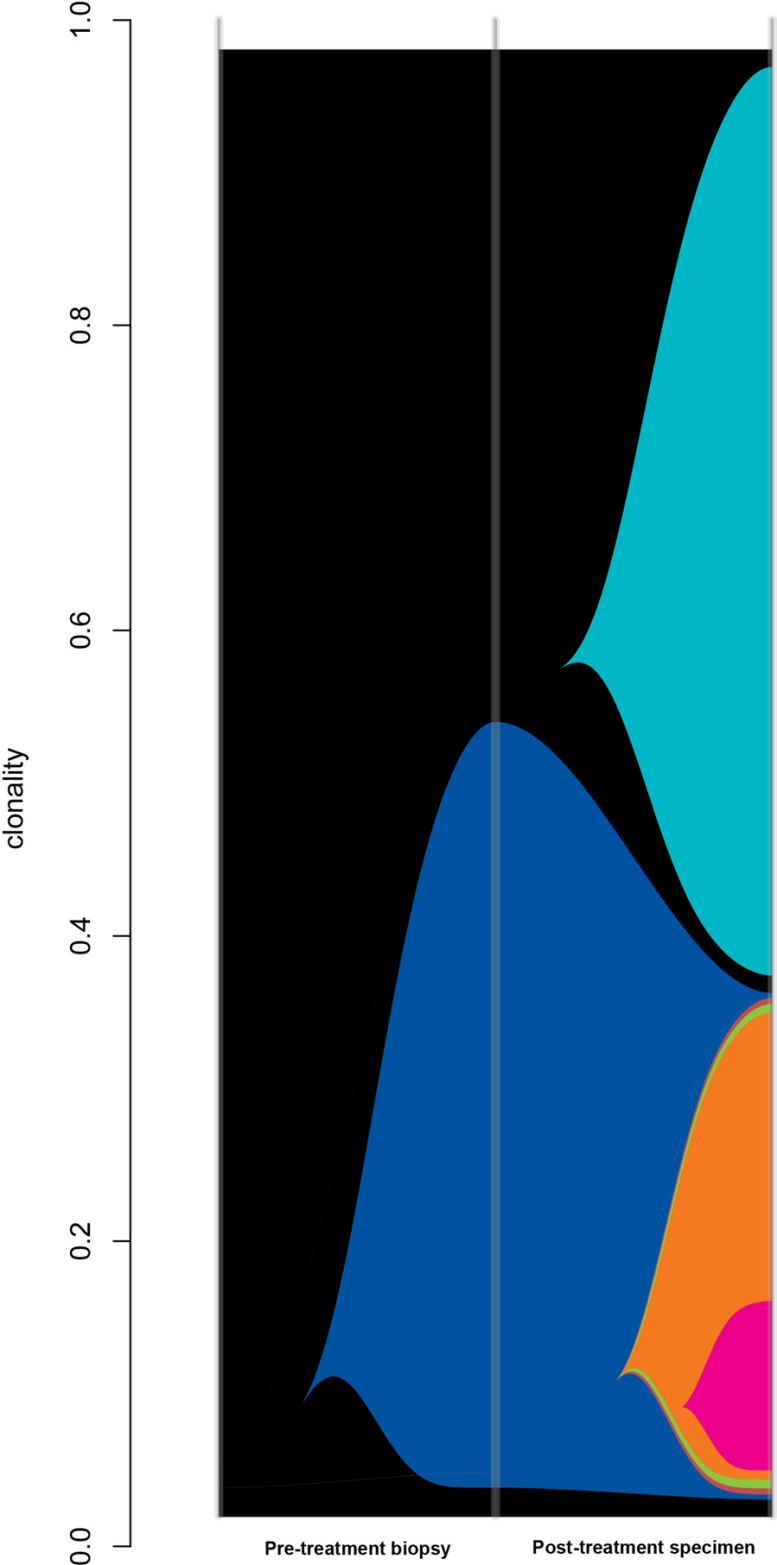
Example river plot of poorly responding cancer; Pre-treatment biopsy demonstrates one clone in sample (clonality 0.57); post treatment specimen shows multiple (five more) clones evolving as a result of radiotherapy selection pressure

**Fig. 5:**
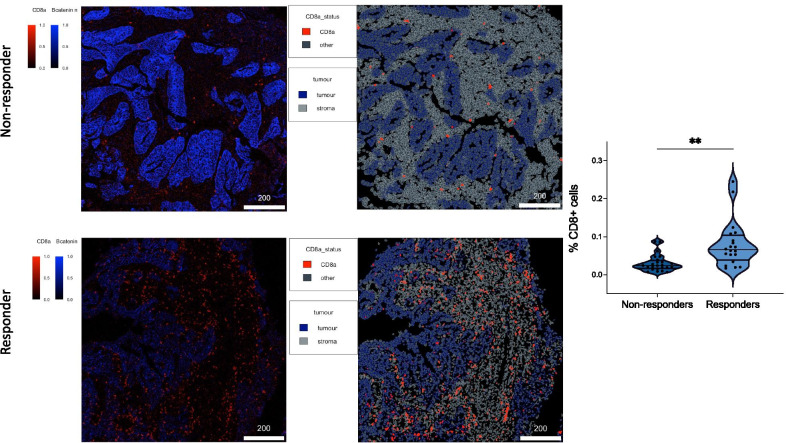
Multi-marker image mass cytometry figures of non-responders (top two images) vs. responders (bottom two images). In left most images, blue staining represents nuclear beta catenin (representing cellular nuclei) and red staining represents CD8 + T-cells. In right most images, blue demonstrates tumour, red demonstrates CD8 + T-cells nad grey demonstrates stoma, showing that the T-cells infiltrate predominantly the stromal compartment. Violin plot to the right shows significant differences between responders and non-responders in terms of percentage of CD8 + T-cells (percentage shown is relative to total number of cells)

In the seven cases from whole trios of normal, pre-treatment biopsy and post-treatment specimens were available (only in the poor response group), clonal evolution analysis with superFreq was carried out. This revealed that subsequent to radiation therapy there was an increase in clonal diversity (Fig. 4) in all patients, suggesting that radiation therapy drove the generation of an increase in clonal diversity. For both the pathCR group and the non-responder group, we calculated MATH score, a measure of tumour heterogeneity for all samples in the pre-treatment biopsies of all samples, finding that there was a median MATH score of 4.7 in non-responders and 4.2 in pathCR group (Mann Whitney p = 0.5036), suggesting that tumour heterogeneity did not play a role in treatment resistance (Fig. 5).

### Methylation analysis

In order to understand if there were epigenetic determinants of response, we compared the methylome of the pre-treatment biopsies of the pathCR group to the tumour of the no response group. This demonstrated 1853 differentially methylated positions and Go:Profiler analysis of these differentially methylated genes demonstrated that the WikiPathways WP306 (Focal adhesion p = 1.16 × 10^−3^), WP185 (Integrin mediated focal adhesion p = 2.42 × 10^−3^) and WP3932 (PI3K-Akt-MTOR pathway p = 3.31 × 10^−2^) were enriched in the pathCR group for demethylation, suggesting potential activation of this pathway. Pathway WP306 contains the PI3K/Akt/MTOR signalling genes as well as WP3932 and represents a general enrichment of this pathway.

Analysis of differentially methylated regions (contiguous blocks of differentially methylated CpGs > 10 in number, DMR) 347 differentially methylated regions. HOMER annotation of top 100 DMRs and then analysis by g:Profiler revealed only enrichment for methylation in the Human Phenotype pathway HP0000104 Renal Agenesis (p = 2.82 × 10^−2^), which contains Wnt signalling and DNA repair genes (ATRX) and the GO:MF term Class II MHC binding (p = 1.77 × 10^−2^).

### Neoantigen prediction

Neoantigen prediction using pVacTools revealed a median of 78 neoantigens per sample (range 9–383) of which the pathCR group had a median 91 neoantigens (IQR 78–154) and the non-responder group a median 74 neoantigens (IQR 28–89, Wilcoxon rank sum p = 0.034).

### DSB pathway and mismatch repair deficiency

Semi-quantitative analysis using QuPath was carried out on white light DAB stained images of the expression of yH2AX, Ku70/80, ATM, MLH1, MSH2 and MSH6 respectively. For the pathCR group the pre-treatment biopsies only were studied and for the non-responder group the post-treatment specimens.

For DNA repair proteins, pathCR cases showed significant differences compared to the non-responder group in the DNA repair associated proteins γH2AX (89% cells expressing vs. 78% cells expressing, p = 0.007), ATM (80% vs. 69%, p = 0.04) and Ku70/80 (88% vs. 29%, p < 0.001), suggesting that a deficiency of normal DNA double strand break proteins was associated with resistance to treatment.

In the mismatch repair associated proteins, pathCR group showed significant differences compared to the non-responder group in MLH1 (70% cells expressing vs. 84% cells expressing, p = 0.001) and MSH6 (76% vs. 88%, p < 0.001). However MSH2 expression (33% vs. 41%, p = 0.10) was not significant. Especially for the mismatch repair proteins, despite there being statistically significant differences, these were only a few percentage points different and little complete loss of expression was seen, suggesting that the role of these proteins is uncertain in this context.

### Imaging mass cytometry analysis

A 40 marker image mass cytometry analysis was performed, and cell counting using CellProfiler determined that with 44 regions of interest across the responders vs. non-responders there were significantly more (proportions as part of total immune cells compartment 7.98% vs. 3.06%, p = 0.0021) CD8 + T-cells infiltrating in tumours with a complete response to radiotherapy than those with a poor response.

## Discussion

In this study, we have demonstrated a number of interesting findings comparing pre-treatment biopsies and post treatment specimens in patients undergoing (nCRT) for rectal cancer.

The most important finding is that in patients achieving a complete or near complete response to nCRT is that the pre-treatment tumour has a high tumour mutational burden [46]. This is because hypermutation within the tumour due to an intrinsic DNA repair defect and leads to increased presentation of neoantigens because of indel and frameshift mutations [47]. This increases immunovisibility and we hypothesise that the increased immunovisibility, coupled with activation of the immune system by irradiation, causes cGAS/STING activation which has been shown to lead to a Type I interferon response [48] leading to migration of immune cells and enhanced regression. Also, it suggests that these patients may benefit from neo-adjuvant anti-PD1/PD-L1 or anti-CTLA4 immunotherapy as high TMB (defined as > 10 muts/mb) has been shown to be correlated with responsiveness to these agents [1]. Another intriguing possibility is that genotoxic therapy, coupled with radiation therapy could be delivered as part of neoadjuvant treatment in the tumour, possibly increasing neoantigen burden and making more patients suitable for immunotherapy [49]. Our sample size of pathological complete responders is relatively small, however we deliberately chose tumour regression where absolutely no cells remained, which is a very rare phenomenon, compared to the phenomenon of “minimal residual disease”, but we believed that this would give a stronger biological signal. We found that this was replicated in a larger cohort (SCORT WS3) although this cohort only underwent limited amplicon sequencing which can serve at best as a proxy for tumour mutational burden.

We have also demonstrated that there is enrichment for mutations in the mTOR/AKT signalling pathways, specifically *PIK3CA* but also as a general trend towards mutations and epigenetic changes in this pathway. *PIK3CA* is a member of the mTOR signalling pathway and makes up the alpha subunit of the PI3K protein. mTOR signalling has previously been highlighted as being of possible relevance in radiosensitivity [50] as a cellular marker of stress in the tumours of patients undergoing chemoradiotherapy. *PIK3CA* signals through the mTORC1/mTORC2 and exerts its downstream effect on AKT [51]. Targeted agents for *PIK3CA* (apitosilib [52]), mTORC1/2 (visusertib [53]) and AKT (MK2206 [54]) exist and are at various stages of clinical development. We suggest that these agents may be utilised as part of a neoadjuvant therapy strategy in order to increase response rates.

*FBXW7*, a gene previously implicated in cell cycle control by ubiquitination? of cyclin-E1 [55] was also significantly enriched for mutations within it in this study. Zhang et al. demonstrated that *FBXW7* also had a role in non-homologous end joining [56, 57], being a binding partner in the complex that repairs damage caused by double strand breaks. Mutations in *FBXW7* may affect its ability to participate in NHEJ and therefore increase radiosensitivity. Mutational signature analysis of the exome sequencing dataset also showed multiple mutational signatures consistent with enrichment in DNA repair, specifically faulty homologous recombination (HR) and nucleotide excision repair. The finding that both *PIK3CA* and *FBXW7* mutations are both enriched in pre-treatment biopsies and found in post-treatment specimens from cancers that do not respond to neoadjuvant therapy means that they may act as biomarkers for lack of response. In oesophageal cancer, induction of novel mutations in cancer driver genes in post treatment specimens has been observed after chemoradiotherapy treatment and our findings may reflect this.

Tumour heterogeneity is a significant problem across all tumours due to drivers that may cause a differential response to therapy because of mutational clonality. Our results show, unsurprisingly, that treatment of samples with neoadjuvant chemoradiotherapy causes an increase in clonal diversity, which may be a driver in the phenomenon of radiation resistance. However, we did not demonstrate any difference between responders and non-responders in terms of MATH score as a measure of tumour heterogeneity, unlike other papers [58]. As we only sampled a single region of tumour, it is also possible the observed lack of difference in heterogeneity is purely due to chance.

The immunohistochemical analysis of tumour samples showed increased yH2AX and ATM expression was associated with response, as well as increased expression of the mismatch repair proteins MLH1 & MSH6. We found this puzzling, as we would have expected the opposite to be true, i.e. loss of expression was needed for response, especially in ATM and yH2AX. However, we hypothesise that these samples may have had regions of loss of normal response and these disappeared as a consequence of response to radiotherapy, or that, is as typical tumour biopsies usually have low tumour content.

Clearly, the best way to understand these defects and investigate them further would be to build a cellular model of rectal cancer in order to modulate these pathways in order to measure responsiveness [59]. Current cell lines have a bias towards their micro environment and although provide reasonable models of single pathway alterations lack the fidelity to measure therapy response when modulated. The ideal model would be an organoid based rectal cancer therapy model [60] as this provides both the 3D structure (enabling cell/cell communication a more representative element of intra-tumoural hypoxia) and more accurate response to therapy, as well as the ability to evolve and resist therapy and the ability to co-culture with other cells in the microenvironment such as T-cells and fibroblasts.

## Conclusions

Therefore, we suggest that based on these findings, a number of factors contribute to the response to neoadjuvant chemoradiotherapy: hypermutation leading to increased neoantigen presentation; enrichment in defects in the mTOR signalling pathway; hypoxia regulated by miR-21-5p and an increase in clonal diversity. Our findings agree with those of Akiyoshi et al. [1] in that increase in neoantigen diversity correlated with response. Kamran [16] however, found no clear molecular defects that predisposed to radioresistance. This could be due to the fact that their analysis was geared towards pathways of treatment resistance rather than those that lead to radiosensitivity.

Our findings suggest a number of new therapeutic avenues for increasing responsiveness to chemoradiotherapy in rectal cancer. We plan to further study these in complex 3D models such as organoids in order to understand whether they will increase response rates when appropriately targeted.

## Supplementary Information

**Additional file 1.** Supplementary data from mutational analysis (MutSigCV, Intogen, Intogen Pathways and COSMIC mutational signatures).

## Data Availability

All data will be uploaded to the SRA upon acceptance of this manuscript.

## Abbreviations

PathCR: Pathological complete response
DNA: Deoxyribonucleic acid
PI3K: Phosphatidylinositol-3-kinase
AKT: AKR thymoma pathway
mTOR: Mammalian target of rapamycin
nCRT: Neoadjuvant chemo-radiotherapy
CCR: Complete clinical response
TEMS: Transanal endoscopic microsurgery
TAMIS: Transanal minimally invasive surgery
DSB: Double strand break
HR: Homologous recombination
NHEJ: Non homologous end joining
TRG: Tumour regression grade
IHC: Immunohistochemistry
FFPE: Formalin fixed, paraffin embedded
HLA: Human leucocyte antigen
MHC: Major histocompatibility complex
CNV: Copy number variant
SV: Structural variant
MATH: Mutant allele tumour heterogeneity
